# Comparison of Acceptable Noise Level Generated Using Different Transducers and Response Modes

**DOI:** 10.1155/2018/3786489

**Published:** 2018-06-26

**Authors:** Liang Xia, Jingchun He, Yuanyuan Sun, Yi Chen, Qiong Luo, Haibo Shi, Yanmei Feng, Shankai Yin

**Affiliations:** ^1^Department of Otolaryngology, Shanghai Jiao Tong University Affiliated Sixth People's Hospital, No. 600, Yishan Road, Xuhui District, Shanghai 200233, China; ^2^Department of Otolaryngology, Shanghai Jiao Tong University Affiliated Xinhua Hospital, No. 1665, Kongjiang Road, Yangpu District, Shanghai 200092, China

## Abstract

The acceptable noise level (ANL) was defined by subtracting the background noise level (BNL) from the most comfortable listening level (MCL) (ANL = MCL − BNL). This study compared the ANL obtained through different methods in 20 Chinese subjects with normal hearing. ANL was tested with Mandarin speech materials using a loudspeaker or earphones, with each subject tested by himself or by the audiologist. The presentation and response modes were as follows: (1) loudspeaker with self-adjusted noise levels using audiometer controls (LS method); (2) loudspeaker with the subject signaling the audiologist to adjust speech and noise levels (LA method); (3) earphones with self-adjusted noise levels using audiometer controls (ES method); and (4) earphones with the subject signaling the audiologist to adjust speech and noise levels (EA method). ANL was calculated from three measurements with each method. There was no significant difference in the ANL obtained through different presentation modes or response modes sound. The correlations between ANL, MCL, and BNL obtained from each two methods were significant. In conclusion, the ANL in normal-hearing Mandarin listeners may not be affected by presentation modes such as a loudspeaker or earphones nor is it affected by self-adjusted or audiologist-adjusted response modes. Earphone audiometry is as reliable as sound field audiometry and provides an easy and convenient way to measure ANL.

## 1. Introduction

The acceptable noise level (ANL) test was developed to quantify the critical amount of background noise that subjects could accept while listening to speech [1–6]. ANL is defined as the lowest signal-to-noise ratio (SNR) that a subject could accept when the target speech was presented at the most comfortable listening level (MCL) [6, 7]. ANL is derived by subtracting the background noise level (BNL) that the subject can accept from the MCL. A low ANL indicates that a subject has a high tolerance for background noises, while a subject with high ANL has low tolerance for background noises [7]. According to Nabelek et al. [8], subjects with an ANL below 7 dB are likely to become successful full-time hearing aid users, while subjects with an ANL above 13 dB are likely to become unsuccessful hearing aids users occasionally or not at all. Previous studies have shown that there is a large variation in ANL across normal-hearing subjects [1, 7, 9–13]. This variation seems unrelated to age [1, 11], gender [14], middle-ear function [15], hearing sensitivity [1, 7, 9, 15, 16], outer hair cell function [15], and efferent pathways utilizing the medial olivocochlear bundle [15]. However, it was influenced by speech materials, noise materials, presentation mode, instructions, and working memory capacity [17].

Several types of sensors have been used to present the ANL signal. In most studies, the sound signal was presented in the sound field through a loudspeaker [18]. However, some studies presented the signals used to measure ANL through an earphone [1]. Olsen and Brännström [17] indicated that the values of ANL obtained from an earphone or loudspeaker may be different. If transducers other than a loudspeaker were used, the ANL data for the specific transducer should be considered. Clinically, audiologists may use ANL data obtained from different transducers. Although most audiologists use a loudspeaker to do ANL, some audiologists could do ANL with earphone when the loudspeaker is not available and predict the ANL results with loudspeaker from the ANL results with earphone. Therefore, it is necessary to contrast different sound presentation modes in ANL tests.

On the other hand, Brannstrom et al. [11] suggested that ANL might be influenced by extrinsic factors such as examiner attitude, instructions, and/or cultural differences in the acceptability of background noise. In most ANL tests, the subject signals the experimenter to adjust the sound volume. However, Nabelek et al. [1] directed subjects to adjust the levels by themselves with visual feedback from the audiometer. The main difference between self-adjusted and audiologist-adjusted measurements is the method to determine the MCL and BNL intensity. During the self-adjusted method, the subjects will determine the MCL and BNL by adjusting the sound levels using the control buttons. During the audiologist-adjusted method, the subjects tell the audiologist the optimal intensity and the maximum intensity signal as the noise level changes continuously; however, there could be a time lag or bias of intensity during this period which can produce differences between the final results and the subjects' true results on ANL tests.

To identify factors that may influence ANL values, this study proposed to investigate and compare the test results of ANL measured through a loudspeaker or earphones and further compared the effect of the self-adjusted or audiologist-adjusted testing method.

## 2. Methods

The program was approved by the Ethics Committee of Shanghai Jiao Tong University Affiliated Sixth People's Hospital. All participants provided written informed consent prior to the study's commencement.

### 2.1. Subjects

This study was conducted in twenty adults (10 male, 10 female) with normal hearing whose native language was Mandarin. Their ages ranged from 21 to 30 years and they were all university students. The criterion for normal hearing sensitivity was pure-tone air conduction thresholds for each ear less than or equal to 15 dB HL at each frequency from 0.25 to 8 kHz with an octave step. The subjects reported no recent otologic problems, such as ear infection, draining ears, otalgia, or surgery on or in the ears during the past eight weeks. The equipment set-ups were calibrated before the study started.

### 2.2. Materials

The test used Mandarin-acceptable noise level material established by Chen et al. [18]. The materials were *The Spring Festival of Beijing* which was chosen from the official textbooks for primary school. The noise signal used to measure BNL was the 12 multitalker babbles routinely used for ANL tests [18]. The ANL signals were a speech signal in one channel and a noise signal in the other channel. In each test condition, the MCL and BNL were tested three measurements, and the average of three measurements for each individual was used as the MCL and BNL of the subjects, and the MCL and BNL of each group were the average values of all the subjects.

### 2.3. Stimuli and Procedure

In sound field audiometry, listeners were tested individually in an audiometric booth that met ANSI standards for ambient noise levels (ANSI, S 3.1-1991). All the stimuli were generated from a compact disc player and delivered via a clinical audiometer (GN Otometrics, Taastrup, Denmark) connected to a calibrated loudspeaker (GSI) located in a sound-proof room. Both speech and noise were presented from the loudspeaker at zero degrees azimuth 1.5 m away from the subjects. The calibration tone was a 1 kHz pure tone. When calibrating, the two channels were calibrated, respectively, and the readout of the VU table on the audiometer panel should be adjusted to 0. The output levels of the speech stimuli and background noise were calibrated at the position occupied by the listener.

In earphone audiometry, listeners were tested individually in an audiometric booth that met ANSI standards for ambient noise levels (ANSI, S 3.1-1991). All the stimuli were generated from a compact disc player and delivered via clinical audiometer (GN Otometrics, Taastrup, Denmark) connected to a calibrated earphone (Sennheiser HDA 200 circumaural earphones). The calibration tone was a 1 kHz pure tone. The output levels of the speech stimuli and background noise were calibrated using B&K 4134 pressure microphone and B&K 4153 simulation ear. When calibrating, the two channels were calibrated, respectively, and the readout of the VU table on the audiometer panel should be adjusted to 0.

### 2.4. The Procedure for Audiologist-Adjusted ANL Tests

Both written and oral instructions were given prior to ANL testing. The instructions were Chinese versions of the English instructions. If the subjects had any doubts, the instructions were clarified. Examples of speech and noise were then presented. Subjects' ANLs were obtained as described previously [18].

The initial stimulus level for each repetition was 30 dB HL for both speech and noise signals, and a 2 dB step size was used for all adjustments for both MCL and BNL. Audiologists increased the sound volume until the speech signal became too loud, then decreased it until it became too soft, and finally the subject selected the sound volume that was the most comfortable listening level. The verbal and written instructions for determining the MCL were as follows:

*You will listen to a story through a loudspeaker or earphones. After a few moments, select the sound volume level for the story that is most comfortable for you, as if listening to a radio. Hand motions will allow you to signal the audiologist to adjust the signal level up (thumbs up) or down (thumbs down) or to stop adjustments (flat palm; this means that you feel the current level is the most comfortable for you).*

Then, BNL was established by adding a noise signal as the speech signal and the subject was instructed to repeat a similar procedure; the speech signal remained fixed at the previously established MCL and the subject increased the sound volume of the noise until it became too loud, then decreased it until the speech became very clear, and finally the subject selected the sound volume that he or she could tolerate without becoming tense or tired while following the speech signal for a long period of time. The subject reported when the BNL had been found. The verbal and written instructions for determining the BNL were as follows [18]:

*You will now listen to the same story with background noise. After you have listened to this for a few moments, select the maximum level of background noise which you would be willing to tolerate without becoming tense or tired while following the story. Hand motions will also allow you to signal the investigator to adjust the signal level.*

### 2.5. The Procedure of Self-Adjusted ANL Tests

The main steps were the same as those of the audiologist-guided test. After brief instructions to the subjects, they conducted the ANL test by themselves. Instructions for the self-adjusted ANL test were as follows:

*You will listen to a story through a loudspeaker or earphones. After a few moments, select the sound volume level for the story that is most comfortable for you, as if you were listening to a radio. You do not need signal the audiologist to adjust the signal level up (thumbs up) or down (thumbs down) or to stop adjustments (flat palm, meaning that you feel the current level is the most comfortable for you). You should regulate the sound volume of sound though the clinical audiometer. When the MCL was found, the multitalker babble was introduced from the loudspeaker or earphones at 30 dB HL. You will now listen to the same story with background noise. After you have listened to this for a few moments, select the maximum level of the background noise which you would be willing to tolerate without becoming tense or tired while following the story. You should regulate the sound volume of the sound though the clinical audiometer too. Both the sound volume level of the 12-talker babble or story are increased in 2 dB steps.*

The MCL and BNL were measured three times with a 30 min gap between two measurements. The ANL from each measurement was obtained by subtracting the BNL from the MCL (ANL = MCL − BNL) for each participant and each experimental condition. The average of the three measurements was used to calculate ANL.

### 2.6. Statistical Analysis

All the statistical analyses were performed using SPSS version 20.0 (IBM Corp., Armonk, NY, USA). Descriptive statistics were calculated for the MCL, BNL, and ANL. The values of different measurements in the same method were compared by paired *t*-test. Pearson's correlation coefficient was used to evaluate the relationship between ANL, MCL, and BNL among three measurements in each condition. Two-way repeated measures analyses of variance (ANOVA) were used to assess the effects of MCL, BNL, and ANL within each method; Pearson's correlation coefficient was used to examine the relationship between the results of ANL and MCL and between ANL and BNL in each method. Correlations between ANL, MCL, and BNL in each method were investigated by Pearson's correlation. The significance level was set at *p* < 0.05.

## 3. Results

### 3.1. MCL, BNL, and ANL Obtained from the Three Measurements with Different Methods

The means and standard error of MCL, BNL, and ANL obtained from the three measurements in different methods are listed in Table 1. The MCL and BNL increased with an increasing number of repetitions in the LS, LA, and ES methods, but the ANL decreased with increasing number of measurements in the LS, LA, and ES methods. However, in the EA method, the MCL and BNL increased first and then decreased with increasing number of repetitions; the ANL decreased first and then increased with increasing number of repetitions. The statistical results comparing the values obtained from the three measurements by paired *t*-tests within each method are shown in Table 2. The significant difference did not exist in most situations except the BNL in the first measurement versus the third measurement with the LS method, ANL in the first measurement versus the second measurement with the EA method, and ANL in the first measurement versus the third measurement with the LS and EA methods.

The correlations of ANL, MCL, and BNL among three measurements within each method indicated that the correlation coefficient of ANL, MCL, and BNL between any two measurements was significant for each method. The range of correlation coefficients was 0.548 to 0.951 with all *p* < 0.05.

### 3.2. MCL, BNL, and ANL Averaged from the Three Measurements with Different Methods

MCL, BNL, and ANL values averaged across three repeated measurements were calculated for the four test methods and were shown in Table 3. A two-way repeated measurement analysis of variance was used to assess the effects of different test methods of on MCL, BNL, and ANL. The dependent variable was MCL, BNL, or ANL. The within-subject factor was response modes, with two levels (self-adjusted or audiologist-adjusted), and the between-subject factor was presentation modes, with two factors (loudspeaker or earphones). First, the results show that the main effect of response modes and presentation modes was statistically significant for MCL (response modes [*F* = 4.364; *p* = 0.043] and presentation modes [*F* = 10.875; *p* = 0.002]). However, the interaction effect of response modes × presentation modes was not significant [*F* = 1.017; *p* = 0.320]. These outcomes indicated that the MCL was affected by response modes and presentation modes. Second, the results show that the main effect of response modes and presentation modes was different in BNL (response modes [*F* = 3.759; *p* = 0.060] and presentation modes [*F* = 8.660; *p* = 0.006]); the interaction effect of response modes × presentation modes was also not significant (*F* = 0.787; *p* = 0.381). The results indicated that the presentation modes may impact BNL, unlike the response modes. The analysis revealed that the main effect of response modes and presentation modes was not statistically significant for ANL (response modes [*F* = 0.191; *p* = 0.665] and presentation modes [*F* = 0.302; *p* = 0.586]); the interaction effect of response modes × presentation modes was also not significant (*F* = 0.008; *p* = 0.931). Both response modes and presentation modes did not affect ANL values. This showed that the ANL values of different response modes do not change with presentation modes because all the differences for interaction effects are not significant.

### 3.3. Correlations between MCL and ANL and between BNL and ANL within Each Method

Pearson correlation coefficients were used to observe the relationship between the MCL and ANL and between the BNL and ANL for each test situation. The correlations between MCL-ANL and between BNL-ANL within each method were not significant: coefficients are shown in Table 4.

### 3.4. Correlations of MCL, BNL, and ANL among Each Method

**Figure 1:**
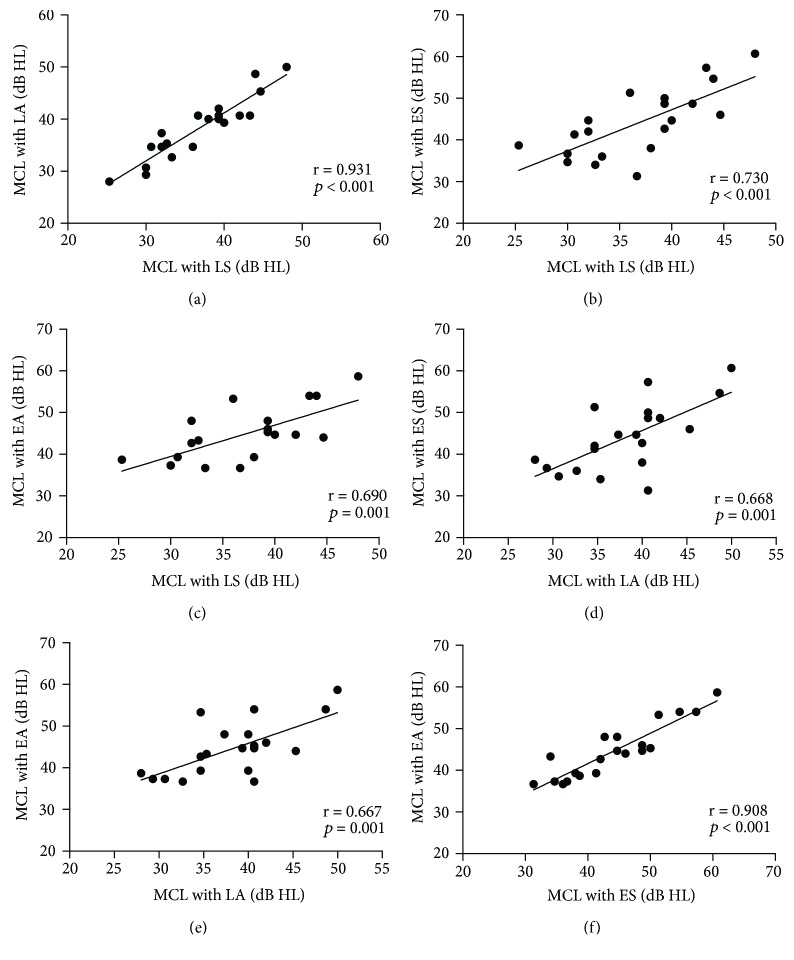
The correlations for MCL between each two methods. A: LS and LA; B: LS and ES; C: LS and EA; D: LA and ES; E: LA and EA; F: ES and EA. The significance level was set *p* < 0.05.

**Figure 2:**
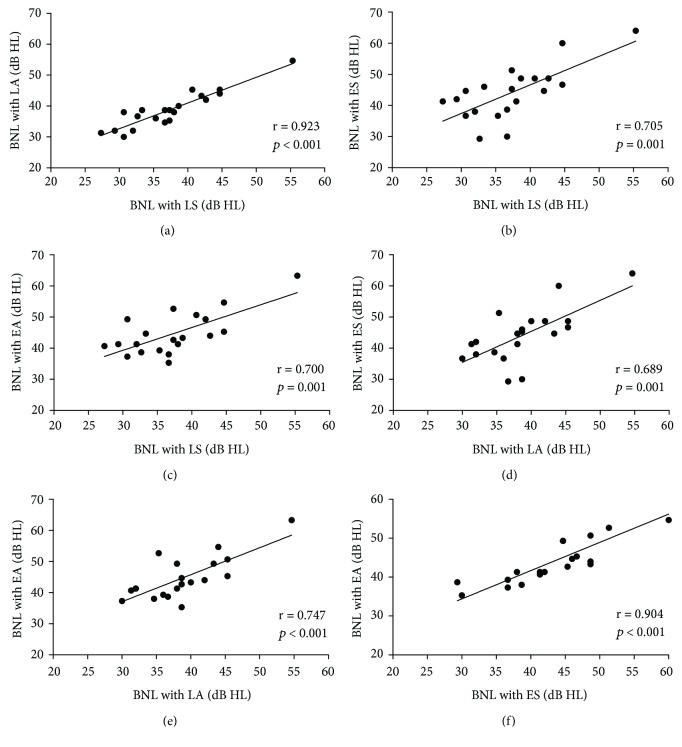
The correlations for BNL between each two methods. A: LS and LA; B: LS and ES; C: LS and EA; D: LA and ES; E: LA and EA; F: ES and EA. The significance level was set *p* < 0.05.

**Figure 3:**
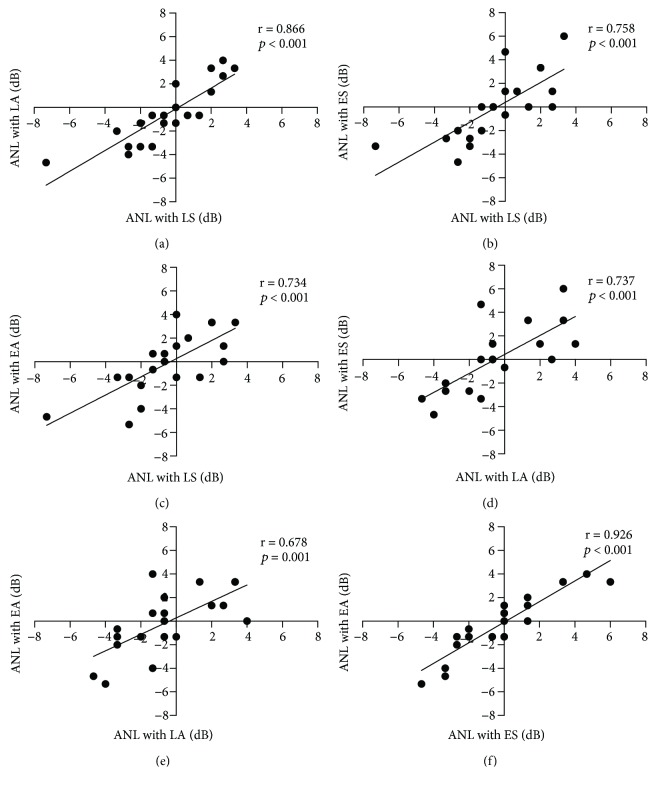
The correlations for ANL between each two methods. A: LS and LA; B: LS and ES; C: LS and EA; D: LA and ES; E: LA and EA; F: ES and EA. The significance level was set *p* < 0.05.

The correlation results of MCL, BNL, and ANL among each method are displayed in Figures 1, 2, and 3, respectively, and all correlations were significant. The correlations of MCL, BNL, and ANL between any two methods were strongly correlated in this study. Correlation coefficients ranged from 0.667 to 0.931; *p* ≤ 0.001.

## 4. Discussion

This study compared the MCL, BNL, and ANL obtained with the LS, LA, ES, and EA methods in subjects with hearing in normal range. The results initially suggest that the values of MCL and BNL increased and the values of ANL decreased with an increasing number of measurements, except in the EA method. Although ANL value tended to decrease, showing a potential learning effect during this test, and tolerance may influence the ANL tests, the difference of MCL, BNL and ANL values obtained from different measurements within each method was not statistically significant in most situations. And our study also shows that the correlations between ANL, MCL, and BNL obtained from both methods and both measurements were significant. This indicated that the repeatability of the ANL test method was credible. Previous studies [18–21] showed that the Pearson correlations suggested significant correlations between ANL and MCL with a LA method, but the correlations of the current study suggested that ANL values were reliable across testing methods; there were no correlations between ANL and BNL or between ANL and MCL in either method. This means that the ANL did not change with MCL or BNL. ANL may be an intrinsic property for every subject and MCL or BNL could affect ANL in different methods.

The main purpose of this study was to compare the acceptable noise level obtained with a loudspeaker or earphones in Chinese subjects with normal hearing. Clinically, the application of the loudspeaker and earphones is different. Sound field audiometry is used to evaluate the auditory function of the subject in a sound field using the loudspeaker. Sound field audiometry has incomparable superiority compared with earphones in children's pure-tone threshold audiometry, hearing aid matching, and evaluation of cochlear implants [22]. When evaluating the effect of the hearing aid, earphones and loudspeakers can be used for the unaided ear, while the patient wearing a hearing aid must be carried out in the sound field through a loudspeaker. Moreover, the results obtained with sound field measurements are more realistic than earphone audiometry. However, sound field audiometry can be easily affected by room size and layout. If the room is too small, subjects may consider it depressing. The capacity for noise isolation and absorption is also important in sound field audiometry. Taken together, the sound field audiometry standards are strict, and the actual operation is complicated. Therefore, it is necessary to conduct ANL testing with earphones and to compare these results with those obtained from earphones and loudspeaker. The results of this study suggest that ANL obtained with earphones is comparable to that obtained with a loudspeaker. Therefore, the hardware requirements and the complexity of the operation could be reduced, which is beneficial to the popularization of ANL tests. For those which do not have sound field conditions and those who are not suitable for sound field audiometry, the ANL obtained from earphones could be used as a reference to that obtained with a loudspeaker in a sound field.

The second main finding of this study compared the acceptable noise level with self-adjusted and audiologist-adjusted levels obtained in Chinese subjects with normal hearing. The ANL tests assess the subject's ability to accept noise, and the researchers speculate that acceptance of noise may be an intrinsic property of an individual [1]. The loci of control and self-control seem to influence acceptable noise levels [23]. The concept of locus of control was proposed by Rotter [24] and refers to a generalized expectation that the results of events are controlled by oneself (internal control) or external forces (external control). The former is the responsibility for the inherent traits of the individual (such as ability and effort), while the latter is the responsibility attributed to factors beyond their own (such as environmental factors and luck). Garstecki and Erler [25] found that the locus of control tends to be external in women, who are less likely to accept the use of hearing aid. Cox et al. [26] reported that personality characteristic had an impact on the assessment of their hearing aid in a subjective questionnaire. Patients with a high locus of external control are less suited to the noise environment. The ANL test results in our study showed that self-adjusted and audiologist-adjusted tests did not differ in the two types of presentation modes. There were also no significant differences in the interactions between the different presentation modes and response modes. Our results indicated that response modes as factors of external control and internal control may not affect the ANL tests, and the locus of control theory is not consistent with our results. This may be because the subjects who participated were not uniform in personality characteristic. If the subjects were grouped according to their personality characteristic, the ANL tests in our study may be different. Future research requires more detailed analysis.

This study has some weaknesses. First, the age of subjects was 20 to 30, so it is not possible to extrapolate the results to all age populations. Moreover, the younger age of the subjects in this study may cause smaller ANL values than seen in other studies. Secondly, the subjects in this study had a good education, and the influence of knowledge level was not studied in previous studies. Thirdly, our results are from subjects with normal hearing. This conclusion remains to be confirmed in the hearing-impaired subjects. Obviously, larger studies are needed to enable multiple variables to be controlled.

However, our research has several protective measures to minimize other potential limitations. When the same audiologist tested three measurements for the subjects, it is impossible for the audiologist to be completely blind to the previous ANL results. Therefore, we attempted to surmount this limitation by ensuring that there was a minimum of a 30 min gap between the two measurements and scheduling the audiologist to perform at least three further measurements on other subjects before repeating the test on that subject again. Moreover, each subject had at least a 30 min gap between each ANL test in each of the four methods, so that the learning effect had less impact on results.

## 5. Conclusion

In conclusion, ANL results obtained through a loudspeaker or earphones with the sound levels adjusted by the subjects themselves or by the audiologist are comparable. The results of correlation relationship for ANL under different methods could provide us the possibility of predicting one measurement based on the other. Clinically, the audiologist could select the appropriate method to conduct the ANL tests according to the facilities and the subjects' conditions.

## Figures and Tables

**Table 1:** The mean and standard error (SE) of MCL, BNL, and ANL obtained from the three repeated measurements in different methods. MCL: most comfortable listening level; BNL: background noise level; ANL: acceptable noise level; LS: measured through loudspeaker with self-adjusted levels using audiometer controls; LA: measured through loudspeaker with the subject signaling the audiologist to adjust the levels of speech and noise; ES: measured through earphones with self-adjusted levels using audiometer controls; EA: measured through earphones with the subject signaling the audiologist to adjust the levels of speech and noise.

Values	Method	1st measurement		2nd measurement		3rd measurement	
		Mean	SE	Mean	SE	Mean	SE
MCL (dB HL)	LS	35.9	1.54	37.2	1.36	37.4	1.37
	LA	38.1	1.61	38.1	1.32	38.6	1.16
	ES	43.3	1.85	43.8	1.83	45.2	2.01
	EA	44.8	1.45	45.2	1.49	43.8	1.65

BNL (dB HL)	LS	35.8	1.73	37.6	1.46	38.5	1.51
	LA	38.2	1.50	38.7	1.42	39.3	1.23
	ES	43.0	2.02	43.9	1.83	45.5	2.21
	EA	44.2	1.39	45.6	1.72	44.2	1.73

ANL (dB)	LS	0.1	0.76	−0.4	0.54	−1.1	0.62
	LA	−0.1	0.72	−0.6	0.56	−0.9	0.62
	ES	0.4	0.83	−0.1	0.49	−0.3	0.84
	EA	0.6	0.56	−0.5	0.78	−0.4	0.55

**Table 2:** The paired t-tests for MCL, BNL, and ANL obtained from the three measurements in different methods. The significance level was set at *p* < 0.05.

Values	Method	1st versus 2nd measurement		1st versus 3rd measurement		2nd versus 3rd measurement	
		*t*	*p* value	*t*	*p* value	*t*	*p* value
MCL (dB HL)	LS	−1.488	0.153	−1.510	0.148	−0.261	0.797
	LA	−0.469	0.645	1.070	0.298	1.889	0.074
	ES	−0.553	0.587	−1.594	0.127	−1.730	0.100
	EA	−0.469	0.645	1.070	0.298	1.889	0.074

BNL (dB HL)	LS	−1.616	0.122	−2.236	0.038^∗^	−1.917	0.070
	LA	−0.592	0.561	−1.291	0.212	−1.301	0.209
	ES	−0.785	0.442	−1.724	0.101	−1.875	0.076
	EA	−1.453	0.163	0.000	1.000	1.759	0.095

ANL (dB)	LS	0.773	0.449	2.698	0.014^∗^	1.437	0.167
	LA	1.045	0.309	1.506	0.148	0.825	0.419
	ES	0.815	0.425	0.941	0.358	0.335	0.741
	EA	2.604	0.017^∗^	2.517	0.021^∗^	−0.252	0.804

^∗^Values were significantly different from each other.

**Table 3:** Values of the mean, standard error (SE), and range for MCL, BNL, and ANL averaged across all the subjects for each method. The significance level was set at *p* < 0.05.

Method	MCL value (dB HL)			BNL value (dB HL)			ANL value (dB)		
	Mean	SE	Range	Mean	SE	Range	Mean	SE	Range
LS	36.83^∗^	1.33	25.33~48.00	37.30^∗∗^	1.47	27.33~55.33	−0.47	0.57	−7.33~3.33
LA	38.27^∗^	1.32	29.33~50.00	38.27^∗∗^	1.32	30.00~54.67	−0.53	0.58	−4.67~4.00
ES	44.10^∗^	1.81	31.33~60.67	44.13^#^	1.91	29.33~64.00	0.00	0.63	−4.67~6.00
EA	44.60^∗^	1.45	36.67~58.67	44.67^#^	1.54	35.33~63.33	−0.10	0.60	−5.33~4.00

^∗^Values of MCL were significantly different from each other. ^∗∗^Values of BNL were significantly different between the ES and EA methods. ^#^Values of BNL were significantly different between the LS and LA methods.

**Table 4:** The correlations between MCL and ANL and between BNL and ANL within each method. The significance level was set at *p* < 0.05.

Method	MCL-ANL		BNL-ANL	
	*r*	*p* value	*r*	*p* value
LS	−0.033	0.890	−0.4173	0.067
LA	0.207	0.381	0.207	0.327
ES	0.038	0.875	0.298	0.202
EA	0.075	0.753	0.325	0.162

## Data Availability

No additional unpublished data are available.
